# Fitness of use of Biopatch® and Tegaderm^TM^ CHG for protecting central venous catheters and arterial lines in critically ill patients

**DOI:** 10.1186/2047-2994-4-S1-O26

**Published:** 2015-06-16

**Authors:** P Eggimann, C Joseph, M-J Thévenin

**Affiliations:** 1 Critical Care Medicine, CHUV, Lausanne, Switzerland; 2 Infection Control, CHUV, Lausanne, Switzerland

## Introduction

Catheter bundles significantly reduce the rate of catheter-associated bloodstream infections. By continuous release of chlorhexidine around the insertion site, the use of antimicrobial devices demonstrated further decrease of the rate of infection below 1 episodes/1000 catheter-days.

## Objectives

To compared the fitness of use of (Biopatch®) and (Tegaderm^TM^ CHG).

## Methods

All central venous catheters and arterial lines, inserted and handled according to a written protocol in all patients admitted to a mixed ICU of 5 units of 7 beds (2000 admissions and 11’000 patients-days yearly) were protected with antimicrobial devices. Biopatch® was used over a 60 month period from 2009. Tegaderm™-CHG was introduced in August 2011 for patients housed in 2 out 5 units and 18 months later replaced Biopatch® in all units. Their fitness of use was compared using a structured questionnaire. The study design did not aim to compare infection rates, which was about 0.3 episodes of infections/1000 catheter days over the period of switch of the devices.

## Results

Health care workers answering the questionnaires were specifically trained to provide care for ICU patients and had followed internal training for catheter handling and care, including specific sessions for the use of antimicrobial devices. Experience captured by the questionnaire run on several tens of individual catheter dressings in all possible insertion sites. Compared to those reported after 60 months of Biotpatch® use (n=24), the overall satisfaction significantly increases after 14 months of Tegaderm™-CHG use (n=42).Categories (in%) very good; good; average, bad increased from 13, 46, 42, 0 to 74, 26, 0 and 0, respectively; p<0.001. This was related to a significant improvement of the ease of installation and of the ability of Tegaderm™-CHG to cover beyond the insertion site protecting in most cases also the area of fixation of the catheter to the skin.

## Conclusion

Based on the significant improvement of fitness of use by the healthcare workers, we decided to replace the Biopatch® by the Tegaderm-CHG™ in the dressing of all central venous catheters and arterial lines for all ICU patients.

## Disclosure of interest

P. Eggimann Grant/Research support from: 3M, Consultant for: 3M, C. Joseph: None declared, M.-J. Thévenin: None declared.

